# Bildung und Bildungsdefizite in Kita und Schule im Rahmen der Coronapandemie

**DOI:** 10.1007/s00112-023-01779-7

**Published:** 2023-06-12

**Authors:** Sabine Walper, Susanne Kuger

**Affiliations:** Deutsches Jugendinstitut e. V., Nockherstr. 2, 81541 München, Deutschland

**Keywords:** COVID-19, Lernen, Kindertagesbetreuung, Distanzunterricht, Lernrückstand, Soziale Ungleichheiten, COVID-19, Learning, Early child care, Distance learning, Learning backlog, Social inequalities

## Abstract

Die Kontaktbeschränkungen während der COVID-19-Pandemie haben die Bildungsteilhabe von Kindern und Jugendlichen massiv eingeschränkt, sowohl im Bereich der Kindertagesbetreuung als auch in den Schulen. Im Lockdown brachen v. a. für junge Kinder vielfach die Kontakte zu Freunden und Kita ab. Sowohl die Qualität der Interaktionen zwischen Fachkräften und Kindern als auch die der Kinder untereinander litt unter der Vielzahl der Schutzmaßnahmen, der hohen Fluktuation der anwesenden Kinder und dem Personalmangel. Pandemiebedingte Gruppenschließungen in der Kindertagesbetreuung betrafen verstärkt Einrichtungen mit einem hohen Anteil von Kindern aus sozial benachteiligten Haushalten, und der Anteil der Kinder mit erhöhtem Förderbedarf stieg. Auch Schulen und Familien waren mit den Herausforderungen unzureichender Digitalisierung konfrontiert. Digitaler Unterricht erreichte häufiger ältere und leistungsstärkere Schüler:innen aus sozial bessergestellten Familien. Die Lernzeit hat sich im Distanzunterricht phasenweise durchschnittlich halbiert und sank v. a. bei fehlendem Kontakt zu den Lehrkräften sowie für leistungsschwache Schüler:innen. Nationale und internationale Trenddaten verweisen auf substanzielle Lernrückstände, insbesondere bei Kindern aus sozial benachteiligten Familien mit geringem kulturellen Kapital und aus zugewanderten Familien, wobei die oft ungünstigeren Lernbedingungen und eingeschränkten Unterstützungsmöglichkeiten in diesen Familien vielfach ausschlaggebend waren. Zudem entfielen im Lockdown außerschulische Erfahrungsräume. In der Post-COVID-Bildungsstrategie sollte stärkeres Gewicht auf das ganzheitliche Wohlergehen der Kinder und Jugendlichen gelegt werden, um den erfahrenen Einschränkungen und Belastungen gerecht zu werden.

## Abstract (en)

The contact restrictions during the coronavirus disease 2019 (COVID-19) pandemic massively limited the educational participation of children and adolescents, both in day care centers and in schools. During phases of lockdown, contact with friends and early child care was often severed, especially for young children. The quality of interactions between staff and children as well as among the children themselves, suffered from the many protective measures, the high fluctuation of children present and the shortage of staff. Pandemic-related group closures in day care centers more severely affected facilities with a high proportion of children from socially disadvantaged households, and the proportion of children with higher support needs increased. Schools and families were also confronted with the challenges of insufficient digitalization. Remote and digital lessons more often reached older and higher performing students from more socially advantaged families. Learning time in distance learning was at times on average halved and decreased especially when there was only little contact with teachers and for low-performing students. National as well as international trend data point to substantial learning gaps, especially among children from socially disadvantaged families with low cultural capital and from immigrant families, with the often less favorable learning conditions and limited support options in these families being the decisive factor. In addition, options for extracurricular activities outside of schools were absent in the lockdown. In the post-COVID education strategy greater emphasis should be placed on the integrated well-being of children and adolescents in order to rectify the constraints and stresses they experienced.

Mit den Kontaktbeschränkungen im Verlauf der COVID-19-Pandemie war über lange Zeit die Bildungsteilhabe von Kindern und Jugendlichen in der Kindertagesbetreuung und Schule gravierend eingeschränkt. Die sich häufig und kurzfristig ändernden Maßnahmen zur Pandemiebekämpfung erschwerten die Arbeit in den Bildungskontexten deutlich und reduzierten das Angebot an Bildungsmöglichkeiten und -aktivitäten. Entsprechend intensiv wird die Diskussion um Lernrückstände von Schüler:innen und Folgen der fehlenden Förderung von Kindern im vorschulischen Alter geführt.

## Hintergrund

Im Verlauf der ersten COVID-19-Infektionswelle waren ab Mitte März 2020 bis August 2020 in allen Bundesländern die Kindertagesstätten und Schulen für die große Mehrheit der Kinder und Jugendlichen geschlossen [29]. Lediglich Absolventenklassen konnten in Präsenz unterrichtet werden. Die im Gegenzug notwendige Umstellung auf Distanz- und speziell Online-Unterricht stieß jedoch auf zahlreiche Hindernisse. Noch im Juni 2020 hatte weniger als ein Drittel der Schüler:innen mehrmals wöchentlich Online-Unterricht im Klassenverbund, weit überwiegend wurde mit Aufgabenblättern gearbeitet [10]. Die Notbetreuung für Kinder, deren Eltern in systemrelevanten Berufen erwerbstätig waren, wurde im Bereich der Kindertagesbetreuung regional sehr unterschiedlich, im Zeitverlauf zunehmend genutzt, blieb jedoch einer Minderheit der Kinder vorbehalten [3, 16].

Obwohl die Schließung der Bildungseinrichtungen schon im Frühsommer 2020 kritisch diskutiert wurde, kam es angesichts steigender Infektionszahlen ab Dezember 2020 bis Ende Mai, teilweise Mitte Juni 2021 zu einem erneuten langen Lockdown, der die Bildungseinrichtungen einschloss. Zunehmend wurde in der Schule auf Wechselunterricht, in der Kinderbetreuung auf „erweiterte Notbetreuung“ oder „eingeschränkten Regelbetrieb“ umgestellt; bei großen bundeslandspezifischen Unterschieden [24]. Auch in der Folgezeit mussten infektionsbedingt viele Kitas und Schulen oder zumindest Gruppen bzw. Klassen (reaktiv) geschlossen werden, wobei die Maßnahmen der Länder und Kommunen unterschiedlichen Regelungen unterlagen. Insofern verlagerten sich das schulische Lernen und die Kinderbetreuung mit kurzer Unterbrechung über lange Phasen hinweg weitgehend in die Familien.

Umso dringender stellt sich die Frage nach dem Ausmaß von Lern- und Entwicklungsrückständen, denen Politik und Fachpraxis begegnen müssen. Neben der Stärke und Verbreitung von pandemiebedingten Bildungsdefiziten unter allen Kindern und Jugendlichen interessiert v. a. deren Ausmaß bei Risikogruppen, die nur in geringem Maße in der Lage waren, wegbrechende Anregungen und Sozialkontakte in institutionellen Bildungsangeboten im privaten Kontext zu kompensieren. Und nicht zuletzt gilt es, die relevanten Faktoren und Prozesse zu identifizieren, die für Bildungsdefizite ausschlaggebend waren. Der vorliegende Beitrag gibt hierzu einen Überblick.

## Frühe Bildung in der Pandemie

### Kitaschließungen im Infektionsgeschehen

Mithilfe des KiTa-Registers konnte ab dem Sommer 2020 ein sehr genaues Bild der Situation in den Einrichtungen der Kindertagesbetreuung in ganz Deutschland gezeichnet werden. Zwischen 2700 und 7000 Kindertageseinrichtungen meldeten wöchentlich bestätigte Infektionen, Schließungen, die Umsetzung von Hygienemaßnahmen und andere pandemiebezogene Merkmale [15]. Die Daten belegen, wie die verschiedenen Infektionswellen den Alltag in den Einrichtungen beeinflussten. Von den Infektionen waren zunächst v. a. die Eltern der Kinder und die Fachkräfte betroffen. Mit sich wandelnden Virusvarianten sowie steigender Verfügbarkeit pharmakologischer und nichtpharmakologischer Schutz- und Hygienemaßnahmen (v. a. Impfung Erwachsener) waren auch Kinder, relativ gesehen, häufiger betroffen. Pandemiebezogene Schließungen wurden jedoch im Zeitverlauf seltener ganzen Einrichtungen, sondern zunehmend nur noch Einzelgruppen auferlegt. Besonders bedeutsam sind Befunde, nach denen mehr Infektionen in denjenigen Einrichtungen auftraten, die von einem höheren Anteil an Kindern aus Familien aus benachteiligten Lebenslagen besucht werden (erst während der Omikron-Welle nahm dieser Befund in seiner Bedeutsamkeit etwas ab). Diese Einrichtungen meldeten folglich auch über alle Wellen hinweg mehr Wochen mit Gruppen- oder Einrichtungsschließungen [17].

Deutliche Beeinträchtigungen des Sozialverhaltens der Kinder sind auch das Resultat fehlender Kontakte

Einrichtungsschließungen, Erkrankungen und Eltern, die ihre Kinder so häufig wie möglich zu Hause ließen, führten zu einer hohen Fluktuation der Kinder in den Einrichtungen und gravierenden Unterbrechungen der Sozialkontakte. Im Gegensatz zu älteren Kindern oder Jugendlichen konnten die ganz Kleinen ihren Austausch mit Freunden nicht auf andere Formate (wie z. B. gemeinsames Online-Spielen, Videotelefonie o. Ä.) verlegen. Entsprechend deutlich sind die Befunde dazu, wie sehr Kinder vor der Einschulung v. a. in den Zeiten von Lockdowns ihre Freunde vermissten [15, 16]. Nach Beendigung der Einschränkungen der Sozialkontakte normalisierten sich die Berichte dahingehend zwar, jedoch hatten sich die fehlenden Kontakte zu diesem Zeitpunkt schon in deutlichen Beeinträchtigungen des Sozialverhaltens der Kinder niedergeschlagen [20].

Ein Hauptgrund für die gravierenden Abbrüche der Sozialkontakte während der Lockdowns war auch die geringe Nutzung digitaler Kontaktformate durch die Kitas. Obwohl ein bemerkenswerter Anteil der Kitas ihre Ausstattung mit digitalen Geräten während der Pandemie verbessern konnte und Online-Angebote für die Kinder (selbstgedrehte Videos, aufgezeichnete Videobotschaften, Online-Chat) ausbaute, gab auch im Frühjahr 2021 deutlich weniger als die Hälfte der Kitas an, mit den Eltern oder Kindern digital, d. h. wenigstens über SMS oder Chat-Anwendungen, ggf. sogar über Videotelefonie zu kommunizieren [15].

### Reduzierte Bildungsangebote und steigender Förderbedarf

Die pandemiebedingten Einschränkungen des Alltags hatten von Anfang an einen deutlich nachteiligen Einfluss auf die Bildungsangebote, die in den Einrichtungen umgesetzt wurden. Ausgeprägte Einbußen zeigten sich in Einrichtungen, die besonders stark von Personalmangel betroffen waren und die von besonders großen Schwierigkeiten bei der Bewältigung pandemiebedingter Mehrarbeit berichteten. Sowohl die Qualität der Interaktionen zwischen Fachkräften und Kindern als auch die der Kinder untereinander litt unter der Vielzahl der zusätzlich umzusetzenden Schutzmaßnahmen, der hohen Fluktuation der anwesenden Kinder und dem Personalmangel [11].

Entsprechende Folgen waren bald zu beobachten: Schon im Frühjahr 2022 berichteten die Einrichtungen von deutlich erhöhten Anteilen an Kindern, die jetzt einen höheren Förderbedarf aufwiesen als noch vor der Pandemie [8]. Die Einrichtungen reagierten darauf mit einer Zunahme an besonderen Bildungs- und Fördermaßnahmen v. a. in den Bereichen Sprache, Motorik, sozialemotionale Entwicklung und psychische Gesundheit [15]. Gleichzeitig meldete mehr als die Hälfte der Bundesländer flächendeckende starke Verzögerungen in der regelmäßigen Sprachstandsdiagnostik und Ausfälle in den Schuleingangsuntersuchungen [2]. Auch Sprachbildungs- und Sprachfördermaßnahmen konnten nur in sehr begrenztem Umfang umgesetzt werden. Entsprechend erhöhte sich schon Ende 2021 in den Meldungen einiger Länder der Anteil der Kinder mit diagnostiziertem Sprachförderbedarf [2].

## Schulen und schulisches Lernen im Pandemieverlauf

### Perspektive der Schulen und Lehrkräfte

Aufschluss über die Situation von Schulen, Schulleitungen und Lehrer:innen gibt u. a. das Schulbarometer, das im Pandemieverlauf bis November 2022 fünf Befragungen vorgelegt hat. Während im April 2020 noch die Probleme mangelnder Digitalisierung und fehlender Vorbereitung auf den Distanzunterricht im Vordergrund standen, traten schon Ende 2020 Probleme der Kinder und Jugendlichen in den Vordergrund [21]. Für fast 90 % der Lehrkräfte war es eine große Herausforderung, die Schülerinnen und Schüler emotional zu unterstützen und zu motivieren bzw. ihnen individualisierte Aufgabenstellungen oder Rückmeldungen zu geben. Selbst im April 2022 stand die Coronapandemie mit ihren Folgen noch an erster Stelle der größten Herausforderungen, die Lehrkräfte nannten, gefolgt vom Lehrkräftemangel, dem schwierigen Verhalten der Schüler:innen und Problemen rund um die mangelnde Digitalisierung von Schulen [22].

Selbst im April 2022 empfanden Lehrkräfte die Coronapandemie noch als ihre größte Herausforderung

Entsprechend eindrücklich schildern Lehrkräfte, mit welchen coronabedingten Problemen ihrer Schüler:innen sie konfrontiert sind. Gut 70 % bekräftigten, dass ihre Schule „trotz aller Bemühungen … aktuell nicht die adäquate Unterstützung beim Lernen bieten“ kann. Hierbei spielen auch die psychischen Belastungen der Schüler:innen eine wesentliche Rolle. Entsprechend rücken rund 60 % der befragten Lehrkräfte die Förderung des psychischen Wohlbefindens der Schüler:innen in der Priorität vor den curricularen Bildungsauftrag. Beiden Aussagen stimmten Lehrkräfte an Grundschulen deutlich häufiger zu als Lehrkräfte an Gymnasien und beruflichen Schulen.

### Lernen im ersten Jahr der Pandemie

Für das Verständnis von Lernrückständen (s. unten) ist es aufschlussreich, in das erste Pandemiejahr zurückzublicken. Einen sehr informativen Überblick über Befunde von 97 Online-Befragungen aus dem Zeitraum 24.03.2020 bis 11.11.2020 aus Deutschland, Österreich und der Schweiz geben Helm et al. [12]. Demnach waren nicht nur Schulen weitgehend unvorbereitet auf den Umstieg auf Distanzunterricht, sondern auch in den Haushalten der Kinder und Jugendlichen stieß dies auf Hindernisse. Bis zu einem Viertel der Schüler:innen und Eltern berichtete eine mangelhafte technische Ausstattung ihres Haushalts für Fernunterricht. Lehrkräfte gaben die Probleme ihrer Schülerschaft noch häufiger an.

Entsprechend schwierig gestalteten sich Kontakte sowie die Teilnahme an verfügbaren Online-Unterrichtsangeboten, erst recht für Schüler:innen mit Förderbedarf [18]. Nur gut die Hälfte der Lehrkräfte erreichte fast alle ihrer Schüler:innen [12]. Umgekehrt berichtete auch nur ein Drittel der Schüler:innen täglichen Kontakt zur Lehrkraft; immerhin rund die Hälfte der Eltern hatte gar keinen Kontakt. Wöchentlichen digitalen Lehrerkontakt/Online-Unterricht hatte ebenfalls nur ein Drittel der Schüler:innen, wobei dies häufiger bei höheren Schultypen und Kindern aus Akademikerfamilien der Fall war [12]. Insbesondere die jüngeren Grundschulkinder hatten nach Befunden einer Befragung des Instituts für Arbeitsmarkt- und Berufsforschung der Bundesagentur für Arbeit (IAB) wenig Kontakt zur Lehrkraft und ungünstige Unterrichtsbedingungen: Auch Aufgabenblätter, Lernvideos bzw. -software und Videokonferenzen wurden im Grundschulbereich seltener eingesetzt als in der Sekundarstufe [1]. Der Mehrheit der Kinder und Jugendlichen fehlte somit die Routine des Unterrichts: Die Teilnahme am digitalen Unterricht war nicht verpflichtend und die Bearbeitung von Aufgabenblättern nicht an eine bestimmte Tageszeit gebunden. Vielfach verlor sich der typische Tagesrhythmus, der sonst durch die Schulzeit vorgegeben war. Gut ein Drittel der Schüler:innen hatte zunehmend Probleme mit dem Aufstehen und der Strukturierung des Tages. Insgesamt zeigte sich ein deutlicher Rückgang der Lernzeit: Bis zu 45 % der Schüler:innen investierten max. 2 Stunden/Tag für Schulaktivitäten [12].

### (Lern‑)Zeit leistungsschwacher und leistungsstarker Schüler:innen

Der Kontakt zur Lehrkraft bzw. die Häufigkeit der unterschiedlichen Unterrichtsformen erwies sich jedoch als wichtig für die Zeit, die die Kinder und Jugendlichen in das schulbezogene Lernen investierten. Insgesamt arbeiteten die in der IAB-Studie befragten Kinder und Jugendlichen während der Schulschließungen durchschnittlich 3,4 h/Schultag für die Schule [1], d. h. nur halb so lange, wie dies Schüler:innen vor der Pandemie taten [31].

Leistungsschwache Schüler:innen reduzierten Lernzeiten stärker als leistungsstarke Schüler:innen

Auch diesbezüglich zeigen sich deutliche Gruppenunterschiede: Leistungsschwache Schüler:innen haben ihre Lernzeiten während der Pandemie stärker reduziert (um 4,1 h) als leistungsstarke Schüler:innen (um 3,7 h) und die gewonnene Zeit zudem vermehrt für nichtentwicklungsförderliche Aktivitäten genutzt ([10], vgl. auch [19]). Die so entstandene Lücke der bildungsorientierten Zeitverwendung zwischen leistungsschwachen und leistungsstarken Schüler:innen war weder durch familienbezogene Faktoren zu erklären, noch wurde sie seitens der Schule oder der Eltern kompensiert. Im Gegenteil erreichte der Online-Unterricht eher leistungsstarke Schüler:innen, die häufiger daran teilnahmen und häufigere individuelle Kontakte zur Lehrkraft hatten.

Über die Erfahrungen von Kindern mit besonderem Förderbedarf oder mit besonderen Einschränkungen liegen nur wenige Ergebnisse vor. Eine Zusatzbefragung zum Nationalen Bildungspanel zeigt, dass Eltern von Kindern mit und Eltern von Kindern ohne besonderen Förderbedarf von vergleichbar vielen Herausforderungen während der Zeiten des Distanzlernens berichten. Allerdings äußern sich Eltern von Kindern mit höherem Unterstützungsbedarf noch weniger positiv über die von der Schule erfahrene Unterstützung und sahen die besonderen Bedürfnisse ihrer Kinder kaum berücksichtigt [18].

### Elterliche Unterstützung

Zudem fehlte zwischen einem Fünftel und einem Drittel der Schüler:innen zu Hause die notwendige Lernunterstützung durch die Eltern oder ältere Geschwister. Eltern waren entweder aufgrund ihrer beruflichen Verpflichtungen (auch im Homeoffice) bzw. anderer Aufgaben, aufgrund fehlenden Fachwissens oder auch aufgrund sprachlicher Barrieren vielfach nicht in der Lage, die nötige Unterstützung zu bieten. Besonders betroffen waren v. a. Eltern mit niedriger formaler Bildung, Eltern mit Migrationshintergrund und Alleinerziehende [12]. Daten des Surveys „Aufwachsen in Deutschland: Alltagswelten“ (AID:A) zeigen weiterhin, dass der Anteil der Eltern, die mit ihren Kindern die Schulaufgaben besprachen und die konkret bei Aufgaben für die Schule halfen, zwischen 2019 und 2021 leicht zugenommen hatte – obwohl die Kinder währenddessen älter und damit auch selbstständiger wurden [2].

Elterliche Unterstützung konnte in der überwiegenden Mehrheit der Fälle nicht ausreichend gewährt werden

Das Problem verschärfend meinte in einer Allensbach-Befragung insgesamt nur ein knappes Fünftel der Eltern, dass sie die Kinder auch von zu Hause aus ganz gut fördern können – Eltern mit einfachem sozioökonomischem Status deutlich seltener (10 %) als Eltern mit hohem Status (29 %) [6]. Vor allem Alleinerziehende machten sich Sorgen, dass ihr Kind mangels Förderung später Nachteile haben könnte. Nach den AID:A-Erhebungen machten sich ebenfalls deutlich mehr Eltern mit niedrigen Bildungsabschlüssen Sorgen über die Bildungskarrieren ihrer Kinder als Eltern mit höheren Bildungsabschlüssen [2]. In der Bildungstrenderhebung 2021 des Instituts zur Qualitätsentwicklung im Bildungswesen (IQB) fielen die rückblickenden und aktuellen Einschätzungen der Eltern zu ihrer Lernunterstützung der Kinder zwar positiver aus, aber auch hier wurden Engpässe und Herausforderungen, nicht zuletzt in der Motivierung der Schüler:innen, deutlich [24].

## Lernrückstände von Schüler:innen

### Trenddaten im Überblick

**Figure 1:**
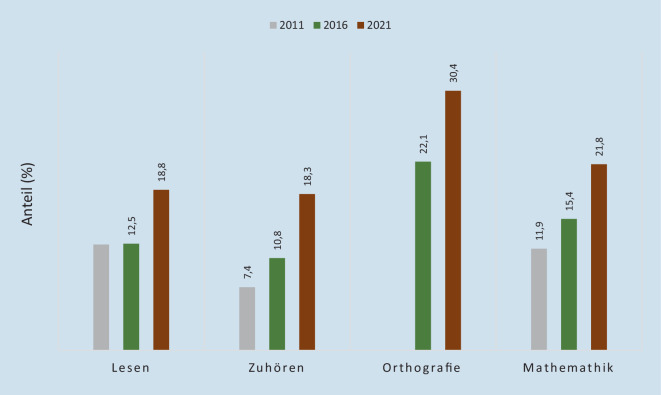

Die IQB-Daten zu Bildungstrends für das Ende der 4. Grundschulklasse zeigen deutlich, wie gravierend die Leistungsrückstände im Verlauf der Pandemie ausgefallen sind [30]. Den Regelstandard erreichten 2021 im Bereich Mathematik nur knapp 55 % der Schüler:innen, und auch in den verschiedenen Bereichen des Deutschunterrichts galt dies nur für gut die Hälfte der Schüler:innen (Lesen: 58 %, Zuhören: 59 %, Orthografie: 44 %). Besonders bedenklich ist die Steigerung der Anzahl jener Schüler:innen, die den Mindeststandard verfehlten. Die Trenddaten für 2011, 2016 und 2021 für die einzelnen Schulfächer in Deutschland insgesamt sind in Abb. 1 dargestellt.

**Figure 2:**
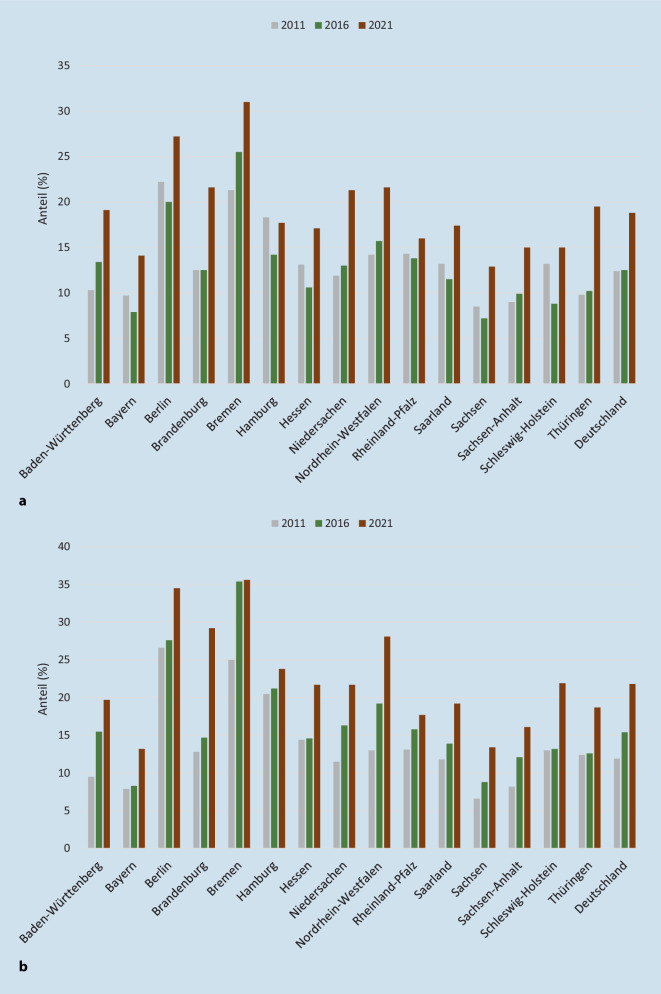

Mit Ausnahme des Lesens hat schon zwischen 2011 und 2016 der Anteil derer, die den Mindeststandard nicht erreichen, zugenommen. (Für die Rechtschreibung fehlen die früheren Daten aus 2011.) Die Zunahme zwischen 2016 und 2021 fiel jedoch noch gravierender aus, insbesondere im Bereich des Zuhörens und der Mathematik, und findet sich beim Lesen überhaupt erst in diesem Zeitraum. Zwischen 18 % und 30 % der Kinder verfehlen diese Mindeststandards. Gleichzeitig bestehen markante Niveauunterschiede zwischen den Bundesländern, mit größten Diskrepanzen zwischen Bayern sowie Sachsen am positiven Ende und Berlin mit Bremen am negativen Ende (Abb. 2a,b). Systematische Unterschiede im Anteil der Förderschüler:innen, der Unterrichtsstunden oder der Ausstattung der Schulen, die dies erklären könnten, lassen sich nicht ausmachen (Kap. 2 der IQB-Studie [25]), aber Kompositionsunterschiede, etwa im Anteil nichtdeutschsprachiger Familien, könnten eine Rolle spielen. Auch die Trends fallen unterschiedlich stark aus, dennoch ist es keinem der Länder gelungen, eine Zunahme von Leistungsdefiziten zwischen 2016 und 2021 zu vermeiden.

Auch Studien aus anderen Ländern verweisen annähernd durchgängig auf negative Effekte der Coronapandemie auf schulische Leistungen. Eine Metaanalyse von 45 Studien aus 15 Ländern ermittelte substanzielle Lerndefizite, die durchschnittlich rund ein Drittel eines Schuljahrs ausmachen und in Ländern mit mittlerem Einkommen stärker ausfielen als in einkommensstarken Ländern [4]. Diese Lernrückstände entstanden früh in der Pandemie und blieben im Zeitverlauf zunächst weitgehend bestehen. Später durchgeführte Erhebungen zeigen also keine Entwarnung im Sinne vollständigen Aufholens der Rückstände an, weder im angelsächsischen Raum noch in den Niederlanden, d. h. jenen Ländern, für die viele Studien verfügbar sind.

Lernrückstände entstanden früh in der Pandemie und blieben im Zeitverlauf zunächst weitgehend bestehen

Teilweise können die Lernrückstände auf die jeweilige Dauer des Lockdowns bezogen werden. Eine große Studie aus den Niederlanden über Daten von rund 350.000 Primarschüler:innen aus 2017 bis 2020 verglich den Lernzuwachs von Schüler:innen, die vor und in der Pandemie getestet wurden, zum Lernzuwachs derer, die ausschließlich vor der Pandemie an den Tests teilnahmen [9]. Während der Pandemie ergab sich ein Leistungsrückstand von rund einem Fünftel eines Schuljahrs. Das entspricht den 8 Wochen, während derer die Schulen in den Niederlanden geschlossen blieben. Eine Studie aus Baden-Württemberg mit vergleichbarem Forschungsansatz berichtet für Fünftklässler nach 2 Monaten Lockdown Lernrückstände von rund einem Monat gegenüber früheren Kohorten in Mathematik und im Lesen [26]. Dass sie hier geringer ausfielen, führen die Autor:innen darauf zurück, dass ihre Daten nicht direkt nach dem Lockdown, sondern im September, also mehrere Monate nach der Wiederöffnung der Schulen mit kompensatorischen Lernangeboten erhoben wurden. Daten aus Hamburg erbrachten ähnliche Ergebnisse [7].

### Bekannte Ungleichheiten steigen

Mit der Verlagerung des Lernens in den häuslichen Kontext war zu erwarten, dass sich die ohnehin bestehenden Kompetenzunterschiede je nach sozialer Herkunft verstärken. Dies entspricht den Befunden der IQB-Bildungstrend-Studie. Während sich die sozialen Disparitäten in den Kompetenzen der Viertklässler zwischen 2011 und 2016 nicht veränderten, stiegen sie 2021 an, wobei v. a. Kinder aus Familien mit geringem kulturellem Kapital den deutlichsten Kompetenzrückstand aufwiesen [23]. Zum Großteil ließ sich dies durch die ungünstigeren Lernbedingungen dieser Kinder erklären (kein Drucker, kein eigener Schreibtisch etc.). Kinder mit Migrationshintergrund mussten ebenfalls größere Lerneinbußen hinnehmen als Kinder ohne Migrationshintergrund [13].

Auch die deutliche Mehrheit der Untersuchungen, die in die internationale Metaanalyse eingingen, zeigen, dass sich Leistungsunterschiede je nach sozialer Herkunft verschärften, sowohl im Lesen als auch in Mathematik, sowohl in der Primar- als auch in der Sekundarstufe und unabhängig davon, wie der sozioökonomische Hintergrund der Kinder erfasst wurde [4]. Ebenso ergaben sich in der Studie aus den Niederlanden deutliche Unterschiede je nach sozialer Herkunft [9]. Schüler:innen aus Familien mit niedrigerer elterlicher Bildung hatten um 40 % stärkere Lerndefizite als der Durchschnitt aller Schüler:innen. Unterschiede je nach Geschlecht, Klassenstufe, Schulfach oder vorherigem Leistungsniveau ergaben sich nicht.

Demgegenüber waren in der Lernstandserhebung Baden-Württemberg das soziokulturelle Kapital und der Migrationshintergrund der Schülerschaft nur von sehr geringer Bedeutung, wurden dort allerdings aggregiert auf Schulebene und nicht für die einzelnen Schüler:innen erfasst. Für leistungsschwache Schüler:innen ergaben sich jedoch etwas stärkere Rückstände im Bereich Mathematik. Vermutlich wird sowohl in der Lernstanderhebung in Baden-Württemberg [26] als auch in der Hamburger Erhebung [7] die Bedeutung sozioökonomischer Merkmale der Schulen unterschätzt, da es in der Coronapandemie größere Ausfälle aufgrund fehlender bzw. nicht auswertbarer Daten speziell bei sozial benachteiligten Schulen gab [7]. So kann ein günstigeres Bild entstanden sein, als es dem Leistungsniveau der Kinder entspricht.

## Außerschulische Erfahrungsräume

Neben und nach der Schule gibt es weitere Lernorte, die für die Entwicklung und das Lernen von Kindern und für ihre Sozialkontakte wichtig sind. Darunter fallen die Ganztagsschulangebote oder der Hort und nonformale Kontexte wie Vereine oder Angebote der Kinder- und Jugendhilfe. Die dort pädagogisch tätigen Personen sind wichtige Vertrauenspersonen, Ansprechpartner und Vermittler im Fall von Problemen und Sorgen sowie Rollenvorbilder für Kinder und Jugendliche. Schulsozialarbeiter:innen, pädagogisch Tätige in Hort und Jugendclub sowie Mitarbeiter:innen der Gesundheitsdienste haben in ihrem engen täglichen Kontakt mit den Kindern und Jugendlichen auch die Möglichkeit, problematische Entwicklungen frühzeitig zu erkennen und Hilfe anzubieten. Von den Maßnahmen zur Eindämmung der Coronapandemie waren allerdings auch Vereine und Angebote der Kinder- und Jugendhilfe betroffen. So erlebten die Sportvereine in Deutschland nach über 2 Jahrzehnten stetiger Zunahme der Mitgliederzahlen einen deutlichen Rückgang der Neuanmeldungen sowie eine übermäßige Anzahl von Vereinsaustritten und verzeichneten in 2021 wieder nur so viele Mitglieder wie im Jahr 2000 [5]. Deutlich weniger Kinder und Jugendliche machten regelmäßig Sport und bewegten sich im sozialen Miteinander. Eltern mit hohem sozioökonomischen Status regen ihre Kinder hierzu deutlich häufiger an und bieten ihnen mehr kulturelle Anregungen [14].

Die Inanspruchnahme öffentlich geförderter Angebote der Kinder- und Jugendhilfe halbierte sich fast

Auch andere Formate der sozialen Teilhabe, Angebote der Kinder- und Jugendhilfe, in denen Kinder und Jugendliche wichtige (Lern‑)Erfahrungen und Schritte der Verselbstständigung machen, wurden deutlich reduziert. Die Zahl der öffentlich geförderten Angebote der Kinder- und Jugendhilfe nahm von 2019 nach 2021 um etwa ein Drittel auf ca. 106.700 Angebote ab. Noch deutlicher reduzierte sich die Anzahl der Teilnehmenden an diesen Angeboten: um ca. 49 % auf nur noch 4,4 Mio. Kinder und Jugendliche (im Jahr 2019 waren es 8,2 Mio.). Unter den vielen angebotenen Formaten wie Jugendclubs, Gruppenstunden und Sportveranstaltungen erlebten v. a. die Ferienfreizeiten, Konzerte, Sportveranstaltungen und Feste einen besonders drastischen Rückgang mit 55 %, nur überboten von der internationalen Jugendarbeit mit einem Rückgang von 74 % der Teilnehmer:innen (und einem Rückgang der Angebote um 64 %, [28]). Diese Erfahrungsräume und Gelegenheiten des sozialen Lernens fehlen Kindern und Jugendlichen. Entsprechend gravierend sind die Folgen im Bereich des Sozialverhaltens und der Selbstregulationsfähigkeit.

## Fazit für die Praxis

- Die pandemiebedingten Einschränkungen im Zugang zu Kindertagesbetreuung und Schule sowie die eingeschränkten Anregungs- und Unterstützungsbedingungen der Familien haben zu substanziellen Lernrückständen von Schüler:innen und einem erhöhten Förderbedarf von jungen Kindern beigetragen.
- Kita und Schule müssen gerade jetzt den Bedürfnissen und Möglichkeiten von Schüler:innen und Lehrkräften besser gerecht werden. Selbstbestimmtes Lernen gehört für Schüler:innen zu den Erfolgsfaktoren guten Unterrichts, wird von ihnen jedoch im Schulalltag immer seltener wahrgenommen.
- Auch im Bildungskontext muss der Fokus auf die ganzheitliche Entwicklung und das Wohlergehen von Kindern und Jugendlichen gestärkt werden, denn Lernen profitiert von körperlicher und psychischer Gesundheit, Selbstwirksamkeit, Lernfreude, Gestaltungsspielräumen und erlebtem sozialen Zusammenhalt [27].
- Beispiele anderer Länder, die – wie Kanada – das Wohlbefinden der Kinder neben hohen Leistungen und Bildungsgerechtigkeit in den Vordergrund ihrer Bildungsstrategie stellen, sollten für Deutschland ein wichtiges Vorbild sein [27].
- Die Einbindung von Kita- und Schulsozialarbeit, Gesundheitsfachkräften an Kitas und Schulen sowie von niederschwelligen außerschulischen Angeboten der Kinder- und Jugendhilfe muss dringend ausgebaut werden.
